# Learning and adaptation in speech production without a vocal tract

**DOI:** 10.1038/s41598-019-49074-4

**Published:** 2019-09-19

**Authors:** Megan M. C. Thompson, John F. Houde, Srikantan S. Nagarajan

**Affiliations:** 1 Department of Radiology and Biomedical Imaging, University of California, San Francisco, CA USA; 2 UC Berkeley-UCSF Graduate Program in Bioengineering, University of California, San Francisco, USA; 3 Department of Otolaryngology Head and Neck Surgery, University of California, San Francisco, CA USA; 4 Department of Radiology and Biomedical Imaging, University of California, San Francisco, CA USA; 5 Department of Speech, Language and Hearing Sciences, Boston University, Boston, MA USA

**Keywords:** Premotor cortex, Motor control

## Abstract

How is the complex audiomotor skill of speaking learned? To what extent does it depend on the specific characteristics of the vocal tract? Here, we developed a touchscreen-based speech synthesizer to examine learning of speech production independent of the vocal tract. Participants were trained to reproduce heard vowel targets by reaching to locations on the screen without visual feedback and receiving endpoint vowel sound auditory feedback that depended continuously on touch location. Participants demonstrated learning as evidenced by rapid increases in accuracy and consistency in the production of trained targets. This learning generalized to productions of novel vowel targets. Subsequent to learning, sensorimotor adaptation was observed in response to changes in the location-sound mapping. These findings suggest that participants learned adaptable sensorimotor maps allowing them to produce desired vowel sounds. These results have broad implications for understanding the acquisition of speech motor control.

## Introduction

Speech production is a complex motor act, with many simultaneous articulations required to translate intentions into vocal output. During speaking, motor areas of the brain generate motor commands that are sent to the muscles of the articulators, causing them to move in a coordinated manner to produce desired speech sounds. How is this remarkable audiomotor skill learned? If we want to study this question, there are several issues that present themselves. First, speaking is generally acquired during early childhood and it is difficult to run extensive, controlled experiments with children. By the time speakers reach adulthood, speech is an overlearned motor act. As a result, investigations of speech learning in adults have generally been confined to examining minor adjustments of speech control. Well-known examples are studies demonstrating sensorimotor adaptation in speaking: the gradual adjustment of speech motor output to anticipate a consistent alteration in sensory feedback^1–10^. While such studies likely reveal important processes involved in the maintenance of correct speech output, they don’t necessarily tell us how the motor skill of speech is initially learned.

Beyond the practical limitations of studying speech learning in adults, a more basic issue is that speech is produced by the vocal tract. This leads to a fundamental question about speech motor learning: how critical are the specific characteristics of the vocal tract (its kinematics, its dynamics, its acoustics, its somatosensory feedback) to the ability to learn to produce speech? Here, we confronted these issues by taking a novel approach to studying how speaking is learned: we examined whether adult speakers could learn to produce speech sounds without using their vocal tract. Instead, they used a touchscreen that generated different vowel sounds whose formants were defined as a continuous function of touch position.

This approach to studying speech motor learning allowed us to examine in particular the centrality of auditory feedback to the process. Auditory feedback is clearly essential to learning vocal speech, as demonstrated by the fact that, in the absence of cochlear implants, children born deaf can rarely be taught to produce more than a few utterances^11^. Further, even after speech is learned, auditory feedback remains important for maintaining speaking skill. Although post-lingual deafened adults can retain intelligible speech for years, many aspects of their speech immediately begin to degrade after deafness^12,13^.

Most models of speech production posit that the influence of auditory feedback on speaking is mediated by audiomotor mappings – learned associations in the speech motor control system between speech motor output and its acoustic consequences^14–16^. These mappings can be learned by remembering the motor commands that successfully produced desired target speech sounds. Furthermore, in many models, these mappings are continuous, allowing for generalization of previously-gained production experience to enable the production of novel speech targets^17^. Once learned, these mappings are assumed to be maintained by comparing the auditory predictions made from the mappings with incoming actual auditory feedback during speaking. Mismatches with predictions can drive immediate corrective motor responses, and consistent mismatches drive update of the audiomotor map. This is called sensorimotor adaptation. Speakers have been shown to exhibit sensorimotor adaptation in response to consistent alterations of the volume^1–3^, pitch^4,5^, and formants^6–8^ of their speech.

In this study, we sought to test the hypothesis that a touchscreen-based vowel formant synthesizer would feasibly allow healthy adult speakers to learn an internal audiomotor map to produce desired speech sounds. Based on this overarching hypothesis we predicted that participants would: (H1) learn an audiomotor mapping that allows them to accurately and consistently produce target speech sounds using the touchscreen, (H2) use this mapping to demonstrate post-learning, accurate production of additional, untrained targets, and (H3) exhibit sensorimotor adaptation of this mapping to accommodate a post-training induced shift of the relationship between touch location and speech sounds produced. To test these hypotheses, we performed two experiments: Experiment 1 sought to address hypotheses 1 and 2 by determining if participants could be trained to learn the correct touch positions needed to produce a range of target vowel sounds based on auditory feedback. It also tested whether this learning would generalize to untrained targets. Experiment 2 investigated hypothesis 3 by examining whether, after training, participants would exhibit sensorimotor adaptation and adjust their touch positions to counteract the effects of a consistent alteration of a learned audiomotor map induced as a shift in formant feedback.

## Results

### Experiment 1: Accuracy and consistency of learned touch responses

**Figure 1:**
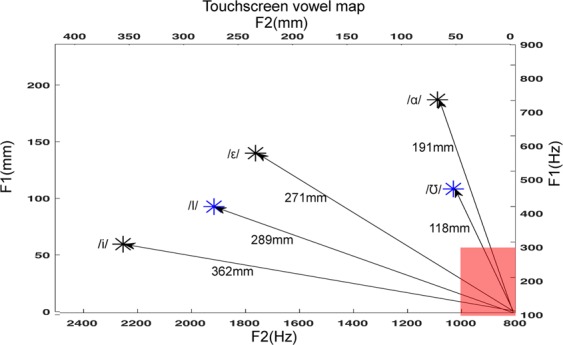
Touchscreen layout including the spatial map dimensions in both Hz (bottom, right) and mm (top, left). Participants’ reach starting position is the lower right-hand corner (red box). Distance from the starting position is calculated for trained (black) and novel (blue) target values (*).

**Figure 2:**
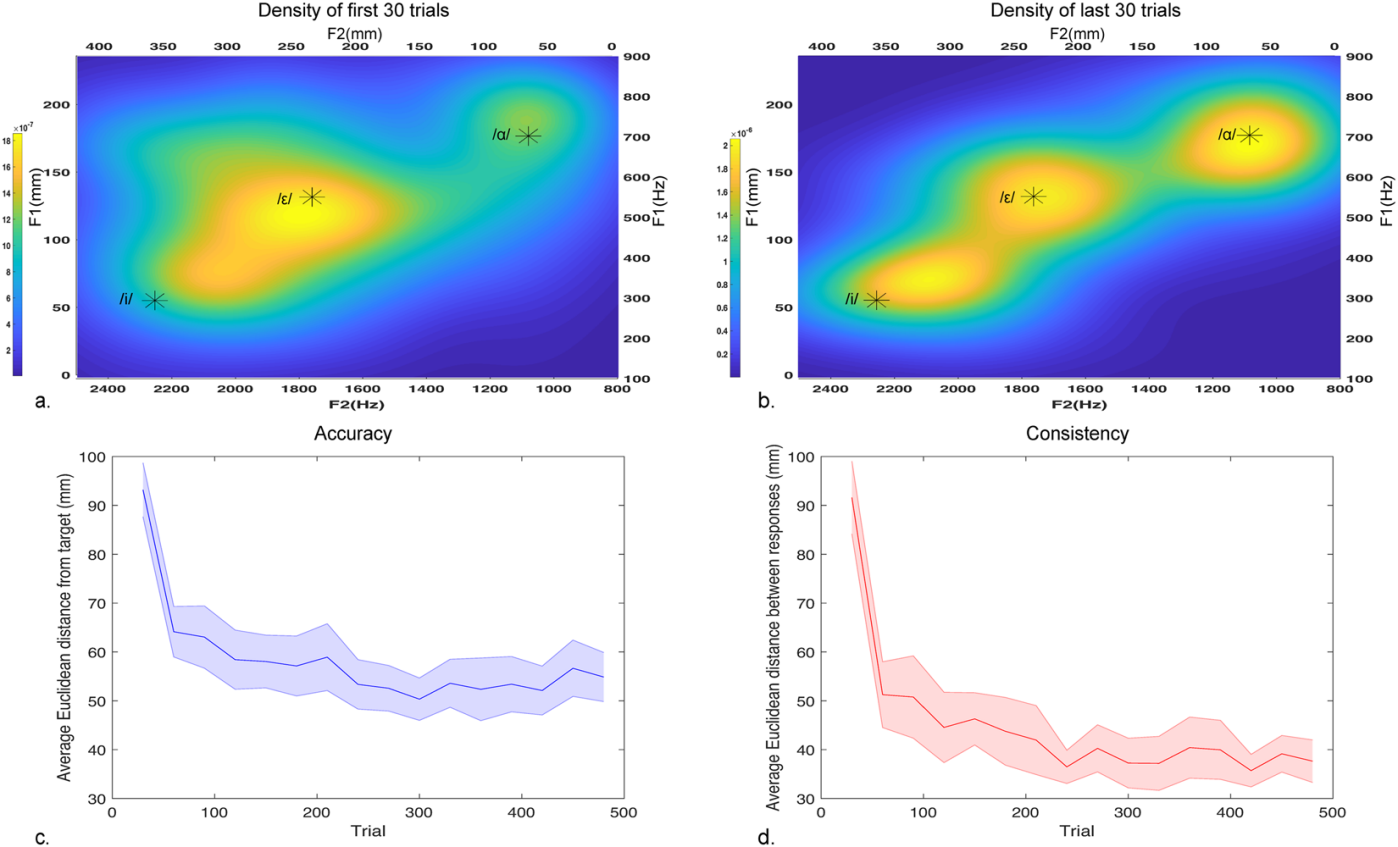
Audiomotor map learning with touchscreen. (**a**,**b**) Response density of all 18 participants during the first 30 trials of training (**a**) and the final 30 trials of the experiment (**b**), with yellow indicating highest response density and blue lowest response density. (**c**,**d**) Improvement on the touchscreen-based vowel production of the task over the course of 480 trials as indicated by response accuracy (**c**) and response consistency (**d**).

Using only auditory feedback, with their eyes closed, participants attempted to repeat auditory vowel targets by reaching to points on a touchscreen that drove a formant synthesizer. Continuous F1 values were determined by y-position and continuous F2 values were determined by x-position (Fig. 1). During the course of a 480-trial training period, participants who performed experiment 1 showed rapid increases in accuracy (Fig. 2c) and consistency (Fig. 2d), evidence supporting our first hypothesis. Accuracy improved significantly (p = 5.70e-15, F = 8.11, 15 degrees of freedom) after the first 30 trials of the experiment and plateaued subsequently (no significant accuracy differences were observed between all subsequent 30-trial windows). Consistency also improved significantly (p = 1.26e-23, F = 12.78, 15 degrees of freedom) after the first 30 trials, and plateaued (no significant differences in consistency were observed between all subsequent 30-trial windows). Many participants demonstrated exponential improvement to a limit, with both accuracy and consistency improving rapidly to a plateau. Density maps for all responses during the first 30 (Fig. 2a) and final 30 trials (Fig. 2b) for all participants visually represent these increases in accuracy and consistency. Sample response densities, accuracies, and consistencies for individual participants can be seen in the supplementary materials (Supplementary Fig. 1). Parameters describing the two-term exponential model $f(x)={A}_{1}{e}^{{\tau }_{1}\cdot x}+{A}_{2}{e}^{{\tau }_{2}\cdot x}$ fitted to each subject’s individual learning curve as a function of trials (indicating increased experience) can also be found in the supplementary materials (Supplementary Table 1).

**Figure 3:**
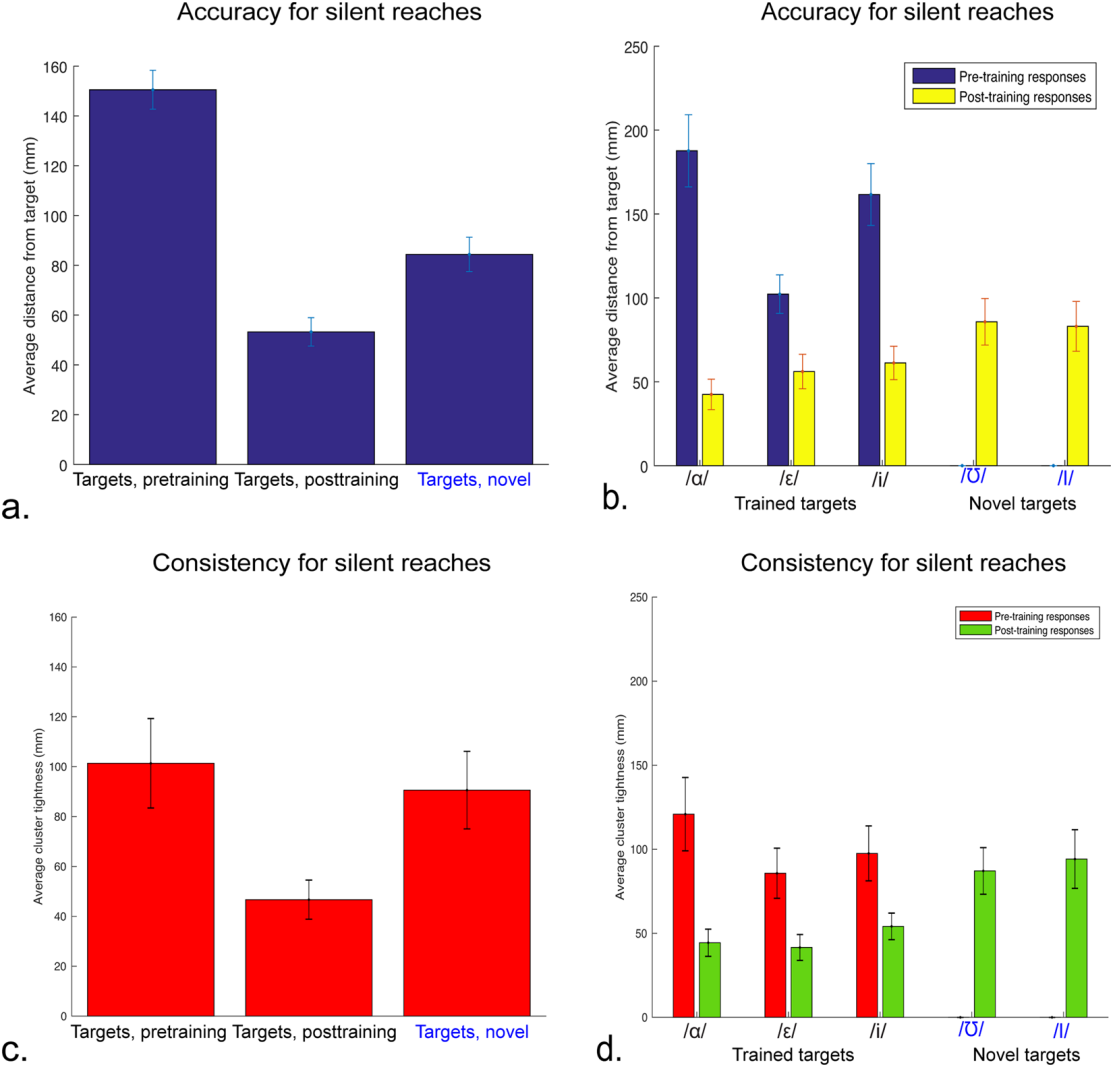
Generalization of audiomotor map learning. Accuracy in silent test trials for reaches to pre-training, trained, and novel targets. (**a**) Left bar: accuracy for training targets, prior to training; middle bar: accuracy for trained targets after training; right bar: accuracy for novel targets after training. (**b**) Accuracy for training targets, prior to training (dark blue) and accuracy for trained and novel targets after training (light yellow). (**c**) Left bar: consistency for training targets, prior to training; middle bar: consistency for trained targets after training; right bar: consistency for novel targets after training. (**d**) Consistency for training targets, prior to training (dark, red) and consistency for trained and novel targets after training (light, green).

Participants demonstrated significant improvements in accuracy even when they received no auditory feedback during open-loop test trials. As shown in Fig. 3a, participants were significantly more accurate in response to trained targets post-training compared to pre-training (p = 1.235e-7, t = 8.653, 17 degrees of freedom), with pre-training accuracy equal to 150.50 mm (±7.82) from the targets, while post-training accuracy was 53.27 mm (±5.70) from the targets. Figure 3b shows that accuracy improved in silent open-loop test trials for all trained targets (p = 1.381e-6, t = 7.240, 17 degrees of freedom): accuracy for /ɑ/ improved from 187.66 mm (±21.50) to 42.48 mm(±9.07); accuracy for /ɛ/ improved (p = 9.443e-4, t = 3.992, 17 degrees of freedom) from 102.23 mm (±11.50) to 56.11 mm (±10.26; accuracy for /i/ improved (p = 2.540e-5, t = 5.7126, 17 degrees of freedom) from 161.61 mm (±18.45) to 61.22 mm (±9.96).

### Experiment 1: Generalization of trained touch responses to novel targets

**Figure 4:**
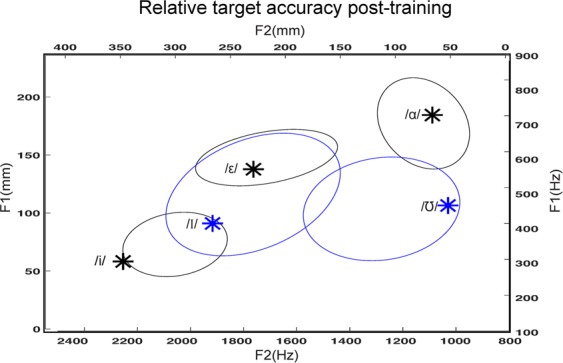
Distribution of post-training responses (ellipses) relative to trained (black) and novel (blue) targets (*). The ellipses are centered on the average F1 and F2 responses. The orientations of the ellipses correspond to the directions of greatest variations in F1 and F2.

Consistent with our second hypothesis, not only did participants rapidly improve on trained targets, they also showed significantly greater accuracy in response to novel targets post-training. Figure 4 visually represents this by showing the most common responses across participants during the open-loop post-training “test” trials without auditory feedback. It expresses the response ranges as ellipses with major and minor axis corresponding to the standard deviations of F2 and F1 values, respectively. These response ellipses center on the average F2 and F1 responses across participants and are subjected to a rotation matrix determined by the maximum F2 and F1 eigenvectors for these data. A similar figure showing most common responses across participants relative to targets in mels, as opposed to Hz, can be found in the supplementary materials (Supplementary Fig. 2). While the linear mapping of (x,y) position to F1 and F2 in Hz results in nonlinear perceptual distortions across the touchscreen, when touch locations were re-calculated in mels the behavior remained qualitatively the same.

As Fig. 4 shows, the post-training accuracy for novel targets, 84.38 mm (±6.89) from the targets, was significantly better than pre-training accuracy on the trained targets (p = 1.368e-6, t = 7.246, 17 degrees of freedom). Responses to novel targets were less accurate than responses to trained targets post-training (p = 5.925e-4, t = 4.2071, 17 degrees of freedom), but there was no significant difference in accuracy between the two individual novel targets (p = 0.840, t = 0.205, 17 degrees of freedom) (Fig. 4b), despite the /ɪ/ target being physically closer to the trained targets (58.46 mm from closest trained target) compared to the /ʊ/ target (75.74 mm from closest trained target) (Fig. 1), as well as /ɪ/ being located roughly between two targets (/ɛ/ and /i/), while /ʊ/ is the rightmost target on the screen.

### Experiment 2: Adaptation of responses to altered feedback

**Figure 5:**
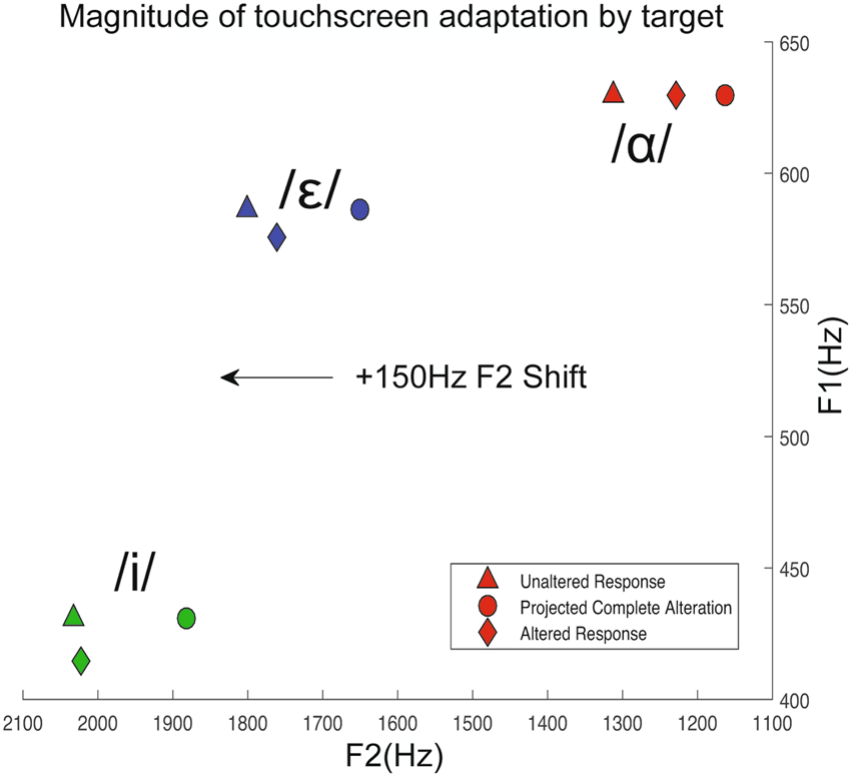
Average adaptation to altered feedback for each target. Triangles indicate average response prior to onset of altered feedback. Circles indicate what projected complete adaptation of −150Hz would be. Diamonds indicate the actual, incomplete average adaptation for each target during the hold phase.

**Figure 6:**
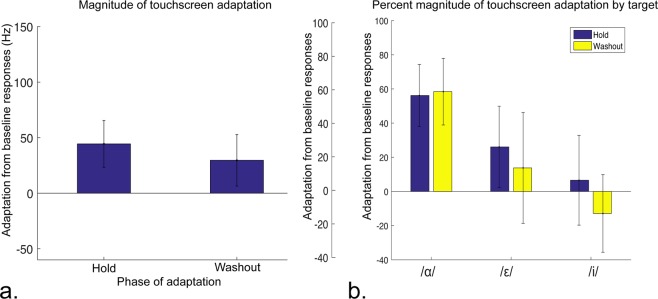
Adaptation of audiomotor map learning. Magnitude of response adaptation to a consistent +150 Hz shift of F2 in participants’ feedback. (**a**) Left bar: response adaptation during the hold phase; right bar: response adaptation during the post-shift washout phase in terms of magnitude of adaptation in Hz (left axis) and percent adaptation (right axis). (**b**) Percent response adaptation during the hold phase (dark blue) and during the post-shift washout phase (light yellow) for each target.

Experiment 2 sought to test hypothesis 3: whether after audiomotor map learning, participants would adapt to a +150 Hz F2 auditory feedback shift. Figure 5 shows the magnitude and direction of the +150 Hz F2 shift (black arrow) during the hold phase. Figure 5 also shows the average baseline responses (triangles) and average hold phase responses (diamonds) compared to the hypothetical complete, −150Hz (100%) adaptation required to achieve the same feedback as pre-shift (circles). During the +150 Hz hold phase, participants significantly adapted their responses compared to the baseline by −44.39 Hz (±34.63) (p = 0.0490, t = −2.111, 18 degrees of freedom) across all targets, an average change of 29.59% (±14.02) (Fig. 6a). During the washout period there was a return to baseline values (p = 0.2168, t = −1.280, 18 degrees of freedom). (Average F1 and F2 responses for all trials for each target can be seen in Supplementary Fig. 4.) For /ɑ/, participants displayed adaptation of 56.59% (±18.15, p = 0.0063, t = −3.0809, 18 degrees of freedom), with post-adaptation percent change amounting to 58.41% (±19.53, p = 0.0078, t = −2.991, 18 degrees of freedom). For /ɛ/, adaptation was 26.05% (±23.85, p = 0.2891, t = −1.092, 18 degrees of freedom) with a post-adaptation period of 6.55% (±32.42, p = 0.6760, t = −0.425, 18 degrees of freedom). For /i/, adaptation was 6.55% (±26.29, p = 0.8060, t = −0.249, 18 degrees of freedom) with a post-adaptation period of −12.88% (±22.75, p = 0.5782, t = 0.566, 18 degrees of freedom) (Fig. 6b). Despite the differences between the adaptation values for each target (only /ɑ/ passed the threshold of significance), a two-way ANOVA revealed no significant difference in adaptation between the three targets.

The magnitude of shift and subsequent level of adaptation for each target expressed in mels can be observed in the supplementary materials (Supplementary Figs. 2 and 3). This shows that there are perceptually different shift magnitudes for each target, yet the resulting adaptation for each target is qualitatively the same as the individual adaptation in Hz.

## Discussion

We investigated the learnability of vowel target production using auditory feedback from a touchscreen with a continuous mapping between location and vowel formants. We consistently found rapid increases in the accuracy and consistency of reaches, generalization to novel targets, and adaptation to feedback changes. Taken together, these results imply the development of an internal, adaptable audiomotor map relating speech sounds with touch locations.

### Development of an audiomotor map: task accuracy and consistency

The development of an audiomotor map is suggested by participants’ rapidly increased accuracy and consistency over the course of the experiment, particularly in the first 60 trials. This map was permanent enough that participants were capable of applying knowledge of trained targets even when they underwent a series of trials in which they received no auditory endpoint feedback. This rapid improvement as a function of experience is visible on a group level, with individual variation in learning rates and experiences visible through the large variation in parameters describing exponential modeling of accuracy improvement (Supplementary Table 1). It is worth further noting that, on average, participants displayed higher consistency than accuracy (Fig. 2): participants’ well-characterized responses did not necessarily converge around the target. This is reminiscent of vocal speech, in which the centroid of individual speakers’ median production varies much more between speakers than within speakers’ production ranges and does not necessarily share the same median for each vowel. This is especially noticeable in the case of the /ɛ/ target, where the most dense area is central relative to the target location. This most frequent response for /ɛ/ is also closer to the arm starting position for each trial, indicating that participants use optimal control to weigh accuracy with effort. However, other factors that contribute to responses converging around points that are not the target are still unclear. Future studies hope to investigate, for instance, whether this convergence location is reflective of an individual’s personal vocal target for the same vowels or because of variations in an individual’s categorical perception of the vowels. Future studies may also investigate the extent to which this audiomotor map may be capable of developing when the auditory targets are not specific to speech. Previous research investigating cross-modality matching have shown the development of a wide array of associations, including scaling sound loudness to light brightness^18^, loudness to vibration and electric shock^19^, and even self-produced loudness to externally generated loudness^20^. Viewing the current study in the context of cross-modality mapping between a touch location and a speech sound, future studies may clarify whether the audiomotor mapping seen in our experiment could be leveraged over participants’ general cross-modality matching capabilities.

Although the link between individuals’ vocal performance and their touchscreen-based vowel production is unclear, auditory or somatosensory perceptual acuity do not appear to be limiting factors that drive accuracy and consistency on this task (note that all experiments were done with the participants’ eyes closed to eliminate confounds from visual feedback). If vowel formant discrimination was a strong limit to the variance of participants’ responses, the size of the ellipsoids centering on each of the vowel targets would be comparable to just-noticeable distances between vowels^21^. However, just-noticeable distance between vowels along the F2 axis in Kewley-Port & Goodman ranged from 15.8–65.7 Hz, much smaller than the range of F2 values observed in our study (178.20–197.49 Hz). Similarly, if somatosensory perceptual acuity were a limiting determinant of performance, the variance in reaches surrounding the target locations observed in our study would have magnitudes similar to variance of reaching target errors in blindfolded subjects. However, a previous study showed that blindfolded controls had endpoint reach errors of ~1.5 cms^22^, much smaller than deviations in the vertical and horizontal dimensions observed in this study (5.71 cm and 2.30 cm respectively). Variance in responses observed in this study is therefore contributed by additional factors and not limited by auditory or somatosensory perceptual abilities.

Instead we propose that accuracy is limited by participants’ ability to develop an accurate location-to-vowel internal map during the relatively short time course of this single experimental session. Such a map would depend on individual weighting of both auditory and somatosensory feedback to guide their reaches. As Lametti, Nasir, & Ostry^23^ showed in vocal speech, different participants weigh somatosensory and auditory feedback differently. If this can be extended to our experiment, participants likely weigh both auditory endpoint and proprioceptive feedback as they attempt to learn the audiomotor vowel map. This weighting is possibly unique to each participant and would explain why responses cannot be predicted by somatosensory or auditory perceptual limits alone.

### Development of an audiomotor map: generalization

In Experiment 1, generalization of accuracy improvement of reaches to novel targets after training is strong evidence for the development of a complete touchscreen location-to-vowel internal map. However, complexities of this generalization merit further examination. First, accuracy of the novel target productions may have been assisted by the fact that, prior to novel target testing, participants had already trained on the original three familiar targets. As a result, those three locations have been eliminated as potential locations for the novel targets. In other words, participants may not have known the location of the novel targets, but they knew three places where the novel targets weren’t. However, given the size of the map and the relative accuracy of the responses to the novel targets, it is unlikely that this can account for the effect entirely. Second, additional help in improving subsequent novel target accuracy may have arisen from subjects’ initial sampling of wide areas of the touchscreen workspace. That is to say, in learning the trained targets participants may have, in their initial random movements, gained information on the then-unknown novel targets. To preclude or partially preclude this possibility, we compared the accuracy of reaches during the first 30 trials of training (defined as the mean Euclidean distance from the target for the first 30 trials, based on the trial window after which accuracy plateaued) with reach accuracy in response to the novel targets during the silent test trials post-training. The results indicate only a weak association, yielding a trend with a non-significant positive Pearson’s correlation coefficient (Pearson’s correlation coefficient = 0.44, p = 0.0645). Participants who explored more during the early phases of training were actually marginally less accurate in their later reaches to novel targets – that is, their early exploration did not improve their subsequent accuracy for novel targets. This provides an indication that learning from inadvertent feedback of the novel targets early in the experiment was unlikely. Additionally, as all responses to the novel targets were during the open-loop test blocks, participants never received auditory feedback on novel target trials, making iteratively learning the novel targets virtually impossible. Third, linear interpolation between trained targets is similarly unlikely because, as discussed earlier, while participants performed significantly better on targets that they had trained on repeatedly, there was no significant accuracy difference between the novel targets regardless of Euclidean distance to the nearest trained target. What could explain this pattern of partial generalization? Previous research indicates that generalization occurs in visuomotor adaptation^24–27^ and generalization of adaptation occurs in vocal experiments with altered feedback^6^. Extrapolating from the adaptation generalization in these studies, we would expect the accuracy of producing novel targets to depend on the distance from novel targets to trained targets. Ghahramani & Wolpert^24^ have indicated that responses to altered feedback on a learned visuomotor map stem from a weighted, sigmoidal function of unaltered target location. However, the uniformity of our participants’ accuracy on novel targets suggests that there is some nonlinear “falloff” in map accuracy based on the distance of the novel targets from trained targets: if there were no accuracy falloff, participants’ responses to novel targets would have the same accuracy as responses to trained targets. One possibility is that participants relied on existing mappings between tongue position and vowel sound production and between fingertip location and two-dimensional surface positions. If the participants are utilizing existing knowledge of the relationship between different vowels’ productions, learning a few vowels might enhance their ability to find novel vowel targets. In other words, they might be able to apply training on some touchscreen-produced vowels to other, untrained touchscreen-produced vowels by generalizing the known relationships between vocal vowel productions to a new production space. This explanation would imply not only that a new audiomotor map has developed to relate touchscreen reaches to expected sound output, but that this map may rely on the existing audiomotor map relating vocal tract kinematics with predicted vocal sound output. This would account for the seemingly plateaued accuracy in the novel targets: participants performed no differently on the novel targets in this study regardless of their distance to the nearest trained target. Presumably, a series of targets spanning the distance between these targets and the trained targets would shed light on the nature of such a falloff function and where the suspected plateau occurs. Future experiments will investigate factors affecting this generalization further.

### Development of an audiomotor map: adaptation to persistent feedback alteration

The development of an internalized audiomotor map is further evidenced by participants’ ability to adapt to subtle feedback alterations. Post-experimental conversation indicated that participants generally did not consciously notice the change in the location-to-sound mapping. This is not surprising because the feedback alteration was gradually ramped up to its maximum value, which was relatively small (a +150 Hz increase in F2, corresponding to shifting the entire map only 36.89 mm to the left on the touchscreen, representing only 8.82% of the touchscreen’s width). Nevertheless, participants still adapted, gradually adjusting their reaches to the targets such that F2 decreased by an average of −44.39 Hz, thus partially countering the effects of the +150 Hz F2 feedback alteration. The result suggests that their audiomotor maps were sufficiently accurate to guide the learning of corrective motor actions to re-obtain auditory feedback closer to the desired target.

An important feature of this adaptation was its incompleteness. Feedback alteration studies in visuomotor research, such as variants of the extremely well-characterized prism goggle experiments, are notably characterized by prevalent complete or nearly complete adaptation (summarized by Welch^28^), whereas vocal adaptation to formant-shifted audio feedback during speaking is virtually always incomplete, ranging between 22.5% and 50%^15^. The fact that the degree of touchscreen adaptation is comparable to the level of vocal adaptation seen in other speech adaptation studies raises interesting questions about the nature of incomplete adaptation. It may be that limitations on the perception of vowels (such as the categorical-like “perceptual magnet” effect^29^) reduce salience of formant frequency shifts in altered speech feedback, resulting in reduced drive to adapt vowel production, regardless of whether vowels are produced using a touchscreen or a vocal tract. It is also possible that some of the incompleteness in adaptation is due to the particular design of the touchscreen task here. In this experiment in particular, participants only heard feedback from the endpoint of their movement to a touchscreen position. This is similar to visuomotor adaptation studies where subjects have been restricted to the endpoint of their movement, studies that have also found incomplete adaptation. For example, Song & Smiley-Oyen^30^ found significantly less adaptation in a cursor rotation task with an adaptively rotated target when participants were denied visual feedback of their cursor. However, if we consider endpoint auditory feedback to be analogous to endpoint somatosensory feedback, Barkley, Salomonczyk, Cressman, Denise, & Overvliet^31^ established that somatosensory feedback combined with endpoint feedback is sufficient to drive motor adaptation. Another study^32^ found that adaptation to robotic force field applications during reaches are not only possible but robust in the absence of visual feedback. Future experiments plan to investigate this in the case of touchscreen-produced speech sounds in more detail.

Another important feature possibly accounting for the incompleteness of adaptation is the continued presence of unaltered somatosensory feedback. This phenomenon is not new. In vocal production, there is individual variability in adaptation of vocal production in response to altered auditory feedback and one suggested explanation has been that individuals weigh auditory and somatosensory feedback differently. Evidence for this comes from Lametti, Nasir, & Ostry^23^, in which researchers applied simultaneous auditory and somatosensory perturbations during speech production. In Lametti’s study, there was no consistent dominance across participants with regards to whether they preferentially corrected to auditory or somatosensory perturbation. Extending this argument, our results are consistent with the supposition that the weighting of auditory and somatosensory feedback can differ across individuals for speech production. This would account both for the level of incomplete adaptation seen in our experiment and for the adaptation even though auditory feedback alterations did not extend to the intact somatosensory feedback mapping.

It is also important to note that the developed somatosensory-to-sound mapping seen in this experiment is unique to each individual, expressed in egocentric coordinated as opposed to arm-centric coordinates. Participants were instructed to use their dominant hand, meaning that left-handed participants had a different arm trajectory but shared a starting point with right-handed participants. Because participants must return to the same starting point to initiate each trial, this was truly a two-dimensional mapping task. This, combined with the presence of a non-adjustable touchscreen stand, kept the angle of the screen consistent and ensured that posture was essentially the same across participants and therefore was not further investigated. One of our goals in developing a simple, two-dimensional map was to eliminate extraneous degrees of freedom to achieve rapid learning of speech production sounds.

### Caveats and limitations

While F1 and F2 formant frequencies are sufficient to distinguish most vowels^33^, the current setup does not even approach replicating the complexity of vocal speech production. Utterances on the touchscreen contain only standalone, monophthong vowels without consonants, making forming most words impossible. Here, we are limited by the two-dimensional nature of the touchscreen. Consonants rely on components other than F1 and F2 to produce unique sounds, and thus the touchscreen would require extra inputs beyond two-dimensional touch location to make them a feasible addition to the touchscreen, as would vowels heavily dependent on a variable F3. But beyond the practical challenges of adding these additional acoustic factors to the touchscreen, adding additional dimensions to the task for participants to learn would greatly hinder the learnability of the task. Indeed, the need to control many more acoustic factors than just F1 and F2 to produce full speech is a likely reason why the motor production of speech generally takes humans years to master. Thus, adding control of additional acoustic factors beyond F1 and F2 might have made the time to learn the task impractically long for a single-session experiment. Indeed, scientists have been attempting to produce electronically synthesized speech from component sounds as far back as Bell Labs’ Voice Demonstrator, “Voder”^34^, a machine consisting of a series of filters that could be manipulated by a highly trained operator to produce speech sounds. This served as an early demonstration of non-linear mapping between circuit resonance and acoustic output production, albeit one with so many degrees of freedom that it was very difficult to learn and required a highly trained operator. More recently, other studies have produced alternate modalities for adults to learn to produce speech in the absence of the vocal tract. However, these modalities either provided visual feedback to facilitate production^35^ or required extensive, prolonged training to successfully synthesize speech sounds^36^.

In addition to lacking the ability to produce many speech sounds, touchscreen-based vowel productions are currently of fixed length and volume, which is not reminiscent of the continuous and highly variable nature of speech. Future iterations of the task could closer replicate the continuous motor demands of speech by creating a more dynamic touchscreen that would require continued contact for continued vocalization and would update continuously with changing touch location. This would allow for the dynamic vowel production seen in vocal speech and give us the ability to investigate such phenomena as participants’ responses to transient, within-trial feedback perturbations.

A further consideration in our experiments is the nature of how motor goals required to produce correct auditory feedback are represented and learned, for example in muscle activations, joint angles, and endpoint finger positions etc. Our experiments were performed with a fixed touchscreen location with respect to the hand, and with participants’ dominate hand. Future generalization experiments may be used to distinguish between various representations of motor goals such as examining performance with a non-trained hand or at a different touchscreen position relative to the body or by constraining movement degrees of freedom of the hand or arm.

### Conclusions and future directions

The current results show that subjects can learn to generate vowels with a touchscreen using auditory feedback, and demonstrate that many characteristics of speech are still present even when speech is produced by a very different effector system. Many vocal speech behaviors, such as generalization of knowledge of some vowels’ production to the production of other vowels and auditory feedback-based motor adaptation, are present in this environment. Thus, the touchscreen could provide a way to study the distinction between vocal production and language production, and to provide a unique platform for studying the development of language.

A simple means of producing speech without the vocal tract could also provide a tool with diagnostic and therapeutic potential. A variety of speech production disorders are linked to production difficulties involving the vocal tract, including, but not limited to, cerebral palsy, aphasia, apraxia, and dysarthria. However, in many patients with speech disorders it is not clear whether the source of errors stems from a loss of phonological abilities, a loss of motor control, or both. For instance, the primarily phonological production errors seen in patients with early-stage logopenic variant primary progressive aphasia may be behaviorally difficult to distinguish from the motor production errors typically seen in nonfluent variant primary progressive aphasia. Similarly, strokes can cause widespread speech damage that may make it difficult to determine whether production difficulties are specifically tied to vocal tract function or language or auditory processing. It is important to have a means to diagnose these patients rapidly and accurately. A platform that asks individuals to produce speech sounds without the vocal tract would provide a means of categorizing patients based on phonological, and not vocal tract motoric, abilities. This would help to provide the correct treatment to improve or restore vital communicative function.

## Methods

### Participants

All experimental procedures were approved by the Institutional Review Board at the University of California, San Francisco. All participants provided written informed consent for their participation in this study. All procedures described were performed in accordance with the relevant institutional guidelines and regulations.

Eighteen healthy participants (8 female, aged 18–43, mean: 26.4, std: 6.65, 0 left-handed) participated in Experiment 1 and nineteen different healthy participants (11 female, aged 18–46, mean: 26.6, std: 8.56, 2 left-handed) participated in Experiment 2. Inclusion criteria for both experiments were: (1) participants had no initial knowledge of the mapping of screen areas to playback sounds, (2) self-reported English as a first language, and (3) had no known speech, language, hearing, learning, or motor deficits (self-reported).

### Apparatus

In this touchscreen-based speech synthesizer, contact with a 41.8 cm by 23.6 cm touchscreen (Surface Acoustic Wave Touch Panel AD-ETP-TS-LEOXXXYYZZZAA, AD Metro, Ottawa, Ontario, Canada) resulted in instant playback of a vowel sound dependent on the location of contact. Vowels were synthesized in real-time in response to users’ touch using a Matlab program adapted from Rabiner and Schafer Vowel Synthesis toolbox^37^ and presented through circumaural headphones. We programmed the touchscreen to control the first (F1) and second formants (F2) of synthesized vowel sounds. The axes of the touchscreen were associated with continuous F2 (800Hz–2500Hz) and F1 (100–900 Hz) formant frequencies, values selected to span even the most conservative F1 and F2 ranges for vowel discrimination in the literature, as summarized by Kewley - Port & Watson^38^. F0, F3, and F4 were fixed at 100 Hz, 2500 Hz, and 4000 Hz, respectively. These values were selected correspond to a clear, intelligible adult male speaker. In this manner, vowel sounds from within a wide formant range could be synthesized in real time in response to the participant touching different locations on the screen. The three experimental targets consisted of /ɑ/ (F1:702 Hz, F2:1089 Hz), /ɛ/ (F1:551 Hz, F2:1763Hz), and /i/ (F1:294 Hz, F2:2254 Hz) and were presented at random with equal frequencies of occurrence with a loudness level of 60 dB and a duration of 600 ms. These three target vowels were selected as extremely common English vowels frequently seen in speech alteration experiments that, together, largely span the space of the touchscreen. As vowel production varies highly among speakers, the formant values for our targets were selected to be comparable to mean formant production values for adult, male speakers^39,40^. As targets were selected before the development of the touchscreen-based vowel synthesizer, researchers utilized text-to-speech vowel synthesis developed by the KTH Royal Institute of Technology, Stockholm^41,42^ to test target formant values for synthesis sound quality and clarity and to make minor adjustments as necessary. Participants were instructed to perform the task with their dominant hand. To give a uniform starting position for the reaches, participants initiated a new trial by pressing the lower right-hand corner of the screen (within a range of 52.25 mm × 59 mm) (Fig. 1). When they pressed this “continue” area post-response, a 300 ms “continue” sound (a 1000 Hz synthesized sinusoidal tone) played, followed by a 600 ms delay, a 0–300 ms jitter, and the auditory presentation of the next target. The /ɑ/ target was 191.28 mm, the /ɛ/ target 271.60 mm, and the /i/ target 362.06 mm from the outermost portion of this starting area. Regardless of response vowel, response feedback was presented with the same loudness and duration as the targets, 60 dB for duration of 600 ms. The layout of the training space can be seen in Fig. 1 and two sample performances of targets and responses can be seen in Supplementary Fig. 1a,b,e,f.

During Experiment 1, two novel targets were eventually inserted throughout the final 120 trials, /ʊ/ (F1:450 Hz, F2:1030 Hz, 117.72 mm from starting position) and /ɪ/ (F1:400 Hz, F2:1917Hz, 288.56 mm from starting position).

### Experiment 1

#### Procedures

Participants were instructed to close their eyes for the duration of the experiment to eliminate confounds from visual feedback. Experiment 1 (N = 18) investigated whether participants could be trained to reproduce specific vowel targets based on auditory feedback, and, if so, whether this generalized to improved post-training performance on novel vowel targets. Over the course of 4 trial blocks of 120 trials, participants were asked to repeat one of three randomized auditory vowel targets by touching the screen. For each trial, participants reached from the starting position to a point on the screen in an attempt to reproduce the target sound. Once participants made contact with the screen, reaches were met with feedback at the end of the reach only, with the exception of 24 “silent”, open-loop test trials before training and another 25 “silent”, open-loop test trials interspersed within the last 120 training trials. During these open-loop test trials, there was no auditory endpoint feedback in response to touches.

#### Analysis methods

To test hypothesis 1, we calculated each participant’s accuracy, or average distance from the target in mm, and consistency, or average distance between responses to the same targets. Response accuracy was determined by the Euclidean distance between each target and its associated response, averaged over a series of 30-trial windows. Response consistency was determined by the mean Euclidean pairwise distance between each response and all of the other responses to the same target, again over a series of 30-trial windows.

We tested increases in both accuracy and consistency using one-way ANOVAs between the data points representing the accuracy or consistency for each 30-trial window for each participant. Improvements in accuracy during silent trials without auditory feedback were determined by paired, two-tailed t-tests between the average accuracy of responses to each target for each participant during the pre-training silent test period and the average accuracy of responses to each familiar target for each participant during the silent test period post-training.

To test hypothesis 2, we sought to determine whether learning generalized to improved accuracy on novel targets. To do this, we determined the difference between pre-training accuracy; as determined by mean distance from target for the pre-training targets during the silent test period preceding training; post-training accuracy on trained targets during the post-training silent “test” trials; and post-training accuracy on novel targets during the post-training silent “test” trials. We did this using a two-way ANOVA with pre-training target accuracy, post-training trained target accuracy, and post-training novel target accuracy as factors. Additionally, we further investigated the nature of this generalization by performing paired, two-tailed t-tests between each of the novel targets to determine if novel target location was a factor significantly affecting accuracy.

### Experiment 2

#### Procedures

Participants were instructed to close their eyes for the duration of the experiment to eliminate confounds from visual feedback. Experiment 2 (N = 19) sought to determine whether trained users would adapt to auditory feedback alterations as they do with vocal speech^6–8^. After 270 trials of unaltered training, the map was subjected to a 30-trial “ramp up” period during which the map shifted +5 Hz in the F2 formant direction (corresponding to the horizontal axis of the touchscreen) each trial. This ensured a gradual transition (generally unnoticed by participants, as indicated by post-experiment interview) to the final F2 shift of +150 Hz. This +150 Hz F2 formant shift persisted during the next 120 trials, or “hold” phase. This shift was then followed by a 30-trial “ramp down” period during which the map shifted −5Hz each trial to ensure a gradual return to baseline feedback. The final 30 trials of the experiment were unaltered, referred to heretofore as the “washout” period. This was to determine if responses returned to “baseline”, here defined as the mean response of the final 30 trials preceding the shift. Hold and washout responses were compared to baseline responses to determine if participants displayed adaptation.

Analysis Methods: Adaptation response was determined by comparing the participants’ raw F2 during the hold phase and the washout phase to their average F2 for the baseline period. Percent adaptation was calculated using the following:

1 $${percent}\,{adaptation}\,\,({hold})=100 \% \frac{({mean}\,{baseline}\,{F2}-{mean}\,{hold}\,{F2})}{150\,{Hz}}$$

or:

2 $${percent}\,{adaptation}\,({washout})=100 \% \frac{({mean}\,{baseline}\,{F2}-{mean}\,{washout}\,{F2})}{150\,{Hz}}$$

According to this metric, complete adaptation to the applied shift would correspond to 100% adaptation, while no change in response to the feedback shift would result in 0% adaptation. Significance of adaptation was calculated by performing paired, two-tailed t-tests on the values for percent adaptation across all 19 participants to determine if the distribution of percent adaptation was significantly different than the null hypothesis of 0% adaptation. This analysis was performed both across all targets and for individual vowel targets. Adaptation differences in each target relative to the other targets, as determined by percent adaptation for each target for each participant, were determined using a two-way ANOVA with participant and vowel target as fixed factors.

## Supplementary information

Supplementary Information

## Data Availability

Participant behavioral data is available from the corresponding author upon request.

## Code Availability

Vowel synthesis and data analysis code will be made available on a public online repository upon acceptance of this paper for publication.
