# Supplementary material for: Learning and adaptation in speech production without a vocal tract
Source: Sci Rep. 2019 Sep 19;9:13582. doi: 10.1038/s41598-019-49074-4 (PMC6753102; doi:10.1038/s41598-019-49074-4)
Supplement: Supplementary file 1 — Supplementary Information [file 41598_2019_49074_MOESM1_ESM.docx]

Title: Learning and adaptation in speech production without a vocal tract

Megan Thompson^1,4^, John Houde^2^ *, Srikantan Nagarajan^3^ *

**Supplementary Figures**

**
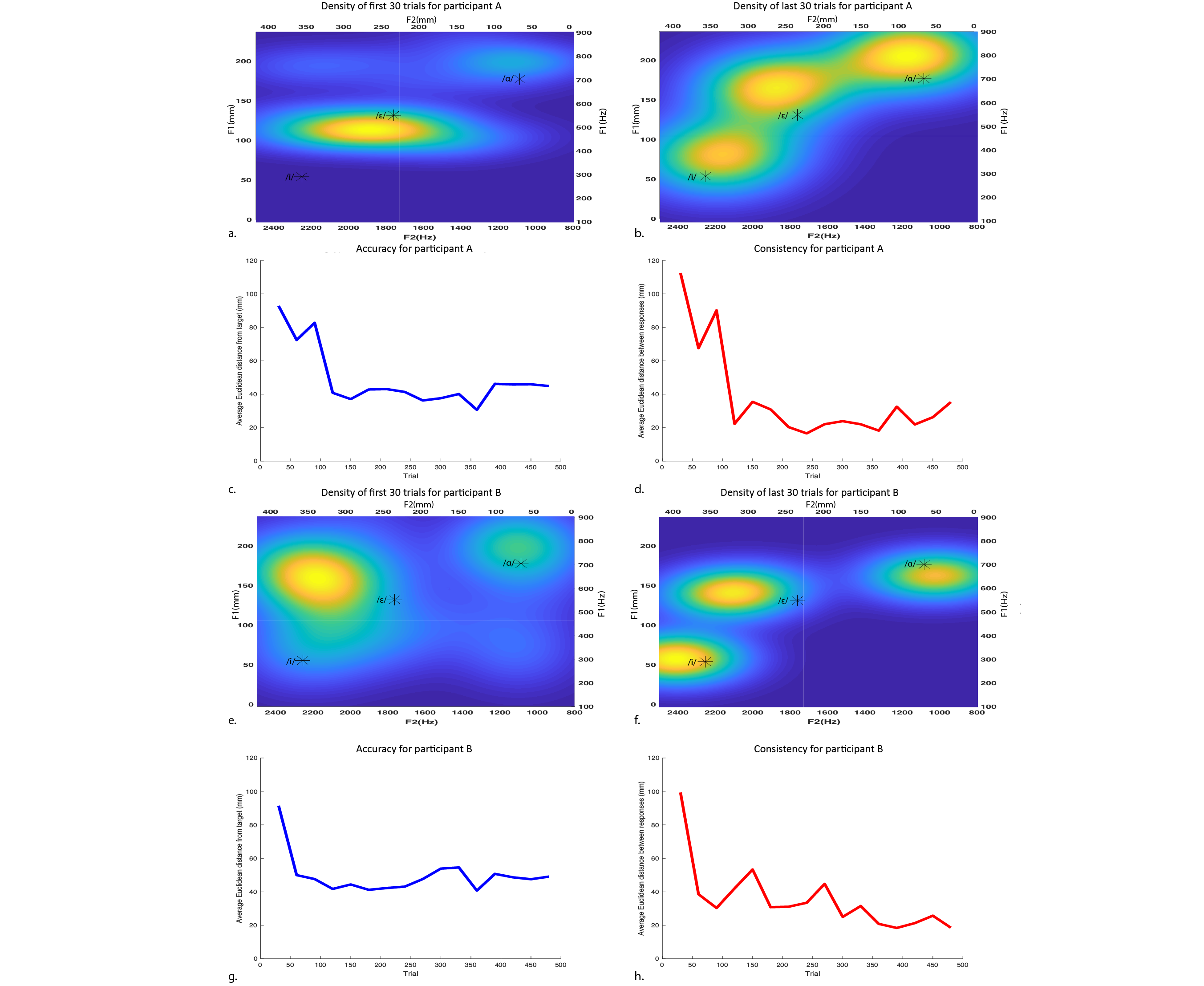
**

**Supplementary Figure 1:** (Color online) Learning in experiment 1 for two individual participants. (a,e): Response density during the first 30 trials of training (b,f): Response density during the final 30 trials of the experiment. Yellow indicates highest response density and blue lowest response density. Improvement on the touchscreen-based vowel production of the task as indicated by (c,g): response accuracy over the course of 480 trials and (d,h): response consistency over the course of 480 trials.


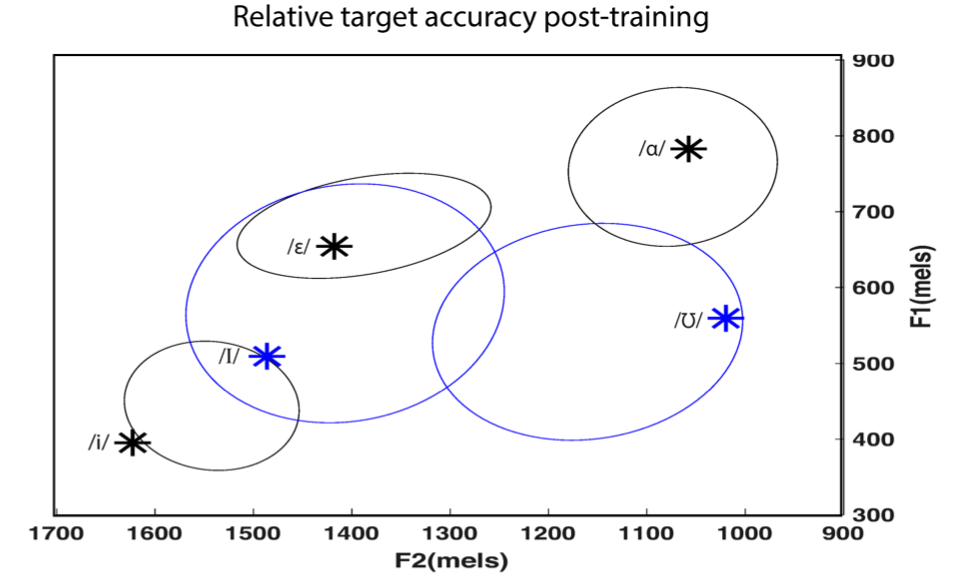


Supplementary Figure 2: (Color online) Distribution of post-training responses (ellipses) relative to trained (black) and novel (blue) targets (*) in mels. The ellipses are centered on the average F1 and F2 responses. The orientation of the ellipses correspond to the directions greatest variations in F1 and F2.


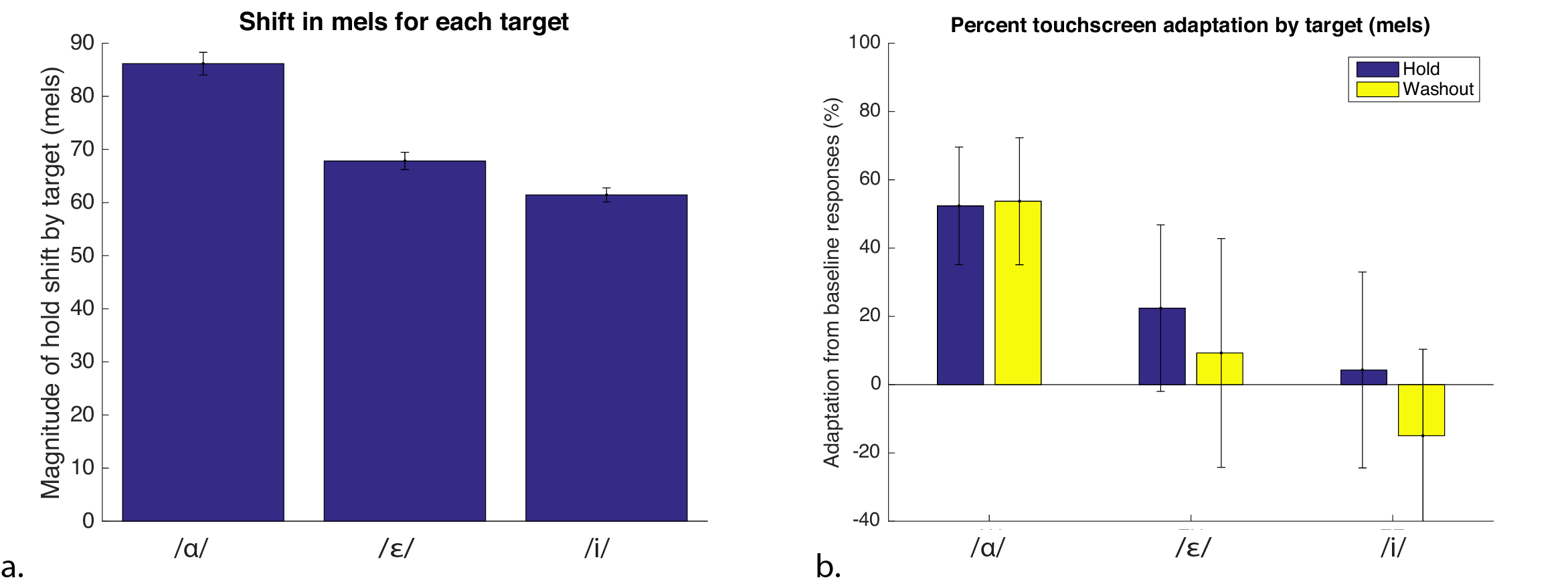


Supplementary Figure 3: (Color online) (a) Magnitude of the F2 shift for each target in mels. (b) Magnitude of percent adaptation compared to baseline during the hold phase (dark) and during the washout phase (light) in mels.


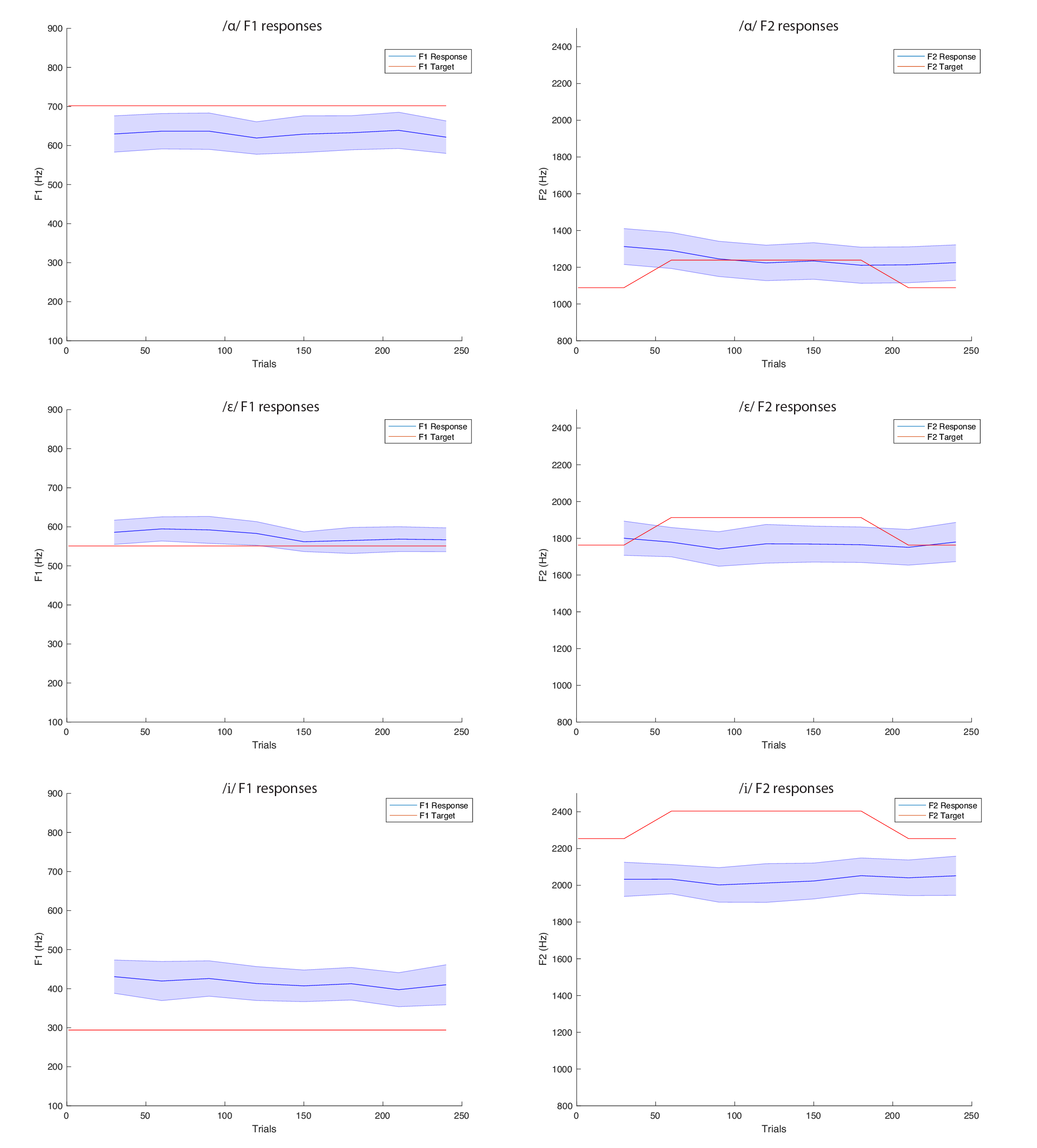


Supplementary Figure 4: Average F1 and F2 responses as a function of trials across all participants during Experiment 2.

Supplementary Table 1: Parameters describing the two-term exponential model fitted to each subject’s individual learning curve $\boldsymbol{f}\left( \boldsymbol{x} \right)\boldsymbol{=}{\boldsymbol{A}_{\boldsymbol{1}}\boldsymbol{e}}^{\boldsymbol{\tau}_{\boldsymbol{1}}\boldsymbol{\cdot x}}\boldsymbol{+}{\boldsymbol{A}_{\boldsymbol{2}}\boldsymbol{e}}^{\boldsymbol{\tau}_{\boldsymbol{2}}\boldsymbol{\cdot x}}$.

| **Participant** | **A_1_** | $\boldsymbol{\tau}$ **_1_** | **A_2_** | $\boldsymbol{\tau}$ **_2_** |
| --- | --- | --- | --- | --- |
| Subject 1 | 146.71 | -490.88 | -98.83 | -155.39 |
| Subject 2 | 105.31 | -43.63 | 41.29 | -2014.79 |
| Subject 3 | 60.29 | -2108.87 | 2053.64 | -8.02 |
| Subject 4 | 40.89 | 2168.07 | 1.58E+12 | -1.22 |
| Subject 5 | 97.83 | -673.5 | 0.01 | 60 |
| Subject 6 | 72.98 | -1400.11 | 0.06 | 76.51 |
| Subject 7 | 66.88 | -3528.18 | 0.27 | 112.22 |
| Subject 8 | 288.83 | -17 | 41.42 | 2511.11 |
| Subject 9 | 6.05E+11 | -1.29 | 28.46 | 929.95 |
| Subject 10 | 30.91 | -121.93 | 29.23 | 970.72 |
| Subject 11 | 119.86 | -1559.68 | 0 | 13.93 |
| Subject 12 | 255.43 | -22.98 | 27.07 | 1098.83 |
| Subject 13 | 75.62 | -168.38 | 38.42 | 8003.22 |
| Subject 14 | 146.2 | -70.88 | 20.99 | 903.68 |
| Subject 15 | 102.73 | -99.08 | 17.56 | 489.2 |
| Subject 16 | 126.66 | -17.94 | 68.65 | -1170.72 |
| Subject 17 | 260.2 | -33.81 | 27.4 | 37320.9 |
| Subject 18 | 1.50E+12 | -1.25 | 78.99 | 1484.7 |
